# Enhancer of Zeste Homolog 2 Induces Pulmonary Artery Smooth Muscle Cell Proliferation

**DOI:** 10.1371/journal.pone.0037712

**Published:** 2012-05-25

**Authors:** Salman A. Aljubran, Ruan Cox, Prasanna Tamarapu Parthasarathy, Gurukumar Kollongod Ramanathan, Venugopal Rajanbabu, Huynh Bao, Shyam M. Mohapatra, Richard Lockey, Narasaiah Kolliputi

**Affiliations:** 1 Division of Allergy and Immunology, Internal Medicine, Morsani College of Medicine, University of South Florida, Tampa, Florida, United States of America; 2 Nanomedicine Research Center and Division of Translational Medicine, Internal Medicine, Morsani College of Medicine, University of South Florida, Tampa, Florida, United States of America; Goethe University, Germany

## Abstract

**Introduction:**

Pulmonary Arterial Hypertension (PAH) is a progressively devastating disease characterized by excessive proliferation of the Pulmonary Arterial Smooth Muscle Cells (PASMCs). Studies suggest that PAH and cancers share an apoptosis-resistant state featuring excessive cell proliferation. The proliferation of cancer cells is mediated by increased expression of Enhancer of Zeste Homolog 2 (EZH2), a mammalian histone methyltransferase that contributes to the epigenetic silencing of target genes. However, the role of EZH2 in PAH has not been studied. In this study, it is hypothesized that EZH2 could play a role in the proliferation of PASMCs.

**Methods:**

In the present study, the expression patterns of EZH2 were investigated in normal and hypertensive mouse PASMCs. The effects of EZH2 overexpression on the proliferation of human PASMCs were tested. PASMCs were transfected with EZH2 or GFP using nucleofector system. After transfection, the cells were incubated for 48 hours at 37°C. Proliferation and cell cycle analysis were performed using flow cytometry. Apoptosis of PASMCs was determined using annexin V staining and cell migration was tested by wound healing assay.

**Results:**

EZH2 protein expression in mouse PASMCs were correlated with an increase in right ventricular systolic pressure and Right Ventricular Hypertrophy (RVH). The overexpression of EZH2 in human PASMCs enhances proliferation, migration, and decrease in the rate of apoptosis when compared to GFP-transfected cells. In the G2/M phase of the EZH2 transfected cells, there was a 3.5 fold increase in proliferation, while there was a significant decrease in the rate of apoptosis of PASMCs, when compared to control.

**Conclusion:**

These findings suggest that EZH2 plays a role in the migration and proliferation of PASMCs, which is a major hallmark in PAH. It also suggests that EZH2 could play a role in the development of PAH and can serve as a potential target for new therapies for PAH.

## Introduction

Pulmonary Arterial Hypertension (PAH) is a hemodynamic condition in which one or more of the pulmonary vasculature is affected and the mean pulmonary arterial pressure and pulmonary vascular resistance are elevated [1] [2], [3]. It afflicts approximately 100,000 individuals and causes 20,000 deaths annually in the United States [4]. PAH is characterized by an excessive proliferation of the Pulmonary Artery Smooth Muscle Cells (PASMCs), the diseased Pulmonary Arteries (PA) in PAH are characterized by vasoconstriction, suppressed apoptosis and increased proliferation within the vascular wall [5], [6]. The underlying causes or genetic factors responsible for the proproliferative and antiapoptotic properties of PASMCs in PAH are not clear [1] [3] [2]. Endothelial dysfunction, an early abnormality in PAH, leads to an increase in vasoconstrictors like endothelin over vasodilators like prostacyclin [1]. Correction of these abnormalities by blockade of the endothelin axis or enhancement of the prostacyclin axis is the basis of the current treatment for PAH; however the morbidity and mortality remains high [7]. More effective therapies for PAH will have to directly and selectively target the mechanisms leading to the proproliferative and antiapoptotic environment in the vascular wall.

Pulmonary hypertension is caused by a variety of factors [8]. The idea that hypoxia alone can cause pulmonary hypertension and significant structural remodeling of pulmonary arteries (PAs) in humans is supported by observations that humans living at high altitudes developed an elevated pulmonary arterial (PA) pressure. Only a small portion is reversible with oxygen [9]. A far greater increase in PA pressure is observed in high altitude versus sea-level residents in response to exercise [8], [9]. Likewise, volunteers were exposed to decreased levels of hypobaric hypoxia over 6-weeks. After 40 days, pulmonary arterial pressures were higher in these hypoxemia subjects [10], [11]. There was a lack of vasodilator response to the acute administration of 100% oxygen in these subjects indicating that significant structural remodeling in the pulmonary vascular bed occurs over this relatively short time [8], [11]. These studies show the importance of chronic hypoxia as a contributing factor in PAH. Therefore, a well-defined hypoxia induced PAH mouse model was utilized in these studies.

Cancer and PAH cells share an apoptosis-resistant and proliferating phenotype [12], and epigenetic gene silencing could be a major factor in the progression of some forms of cancer [13]. Enhancer of zeste homolog 2 (EZH2) is a mammalian histone methyltransferase that contributes to the epigenetic silencing of target genes through methylation and promotes proliferation and metastasis of cancer cells. EZH2 expression and methylation status are known to be predictive of the progression and treatment outcomes of certain cancers [14] [15]. Abnormal methylation has been suggested to be associated with cardiovascular pulmonary diseases [16]. However, until now, the role of EZH2 in cardiovascular and pulmonary diseases has not been documented.

Since excessive cell proliferation is a common feature of PAH and some cancers, it is hypothesized that EZH2 plays a role in the proliferation of pulmonary arterial smooth muscle Cells (PASMCs) in PAH. This report is the first investigation focused on exploring the role of EZH2 in PAH. Our results show that the levels of EZH2 are induced in hypoxia-mediated PAH mice and that overexpression of EZH2 modulates proliferation, migration and resistance to apoptosis of PASMCs, all of which are known to be hallmarks in the pathogenesis of PAH.

## Materials and Methods

### Cells and cell culture

Human PASMCS (HPASMCs) were purchased (Cascade Biologics, Portland, OR) and maintained in growth media M231(Invitrogen, Carlsbad, CA) supplemented with Smooth Muscle Growth Supplement (SMGS) (Invitrogen, Carlsbad, CA) at 37°C in 5% CO_2_ as described in earlier studies [17].

### Hypoxia exposure

Approval of the study protocol was obtained from the Massachusetts General Hospital Institutional Animal Care and Use Committee with protocol number A3596-01. All mice were maintained in a specific-pathogen-free animal facility at Massachusetts General Hospital and all animal experiments were carried out according to the provisions of the Animal Welfare Act, PHS Animal Welfare Policy, and the principles of the NIH Guide for the Care and Use of Laboratory Animals. Five age and sex-matched C57BL/6 mice were exposed to room air (21% FiO2) or hypoxia (10% FiO2) at sea level. All animals were 3 months old and weighed 22.5±2.0 grams. Mice were housed in a sealed chamber, and the O_2_ concentration maintained at 10% by controlling the inflow rate of compressed air and N_2_. The CO_2_ concentration was maintained at <0.4% with a CO_2_ absorbent. Gas samples were tested twice per day during the entire experimental period to monitor O_2_ and CO_2_ tension. The chamber was unsealed for less than 30 minutes twice per week to replenish food, replace CO_2_ absorbent and clean the cages as described [18].

### Measurement of right ventricular systolic pressure (RVSP)

Mice hemodynamic measurements were performed as previously described [18] to assess the right ventricular hypertrophy. The right ventricle was dissected from the left ventricle and interventricular septum was weighed separately, and the weight ratio was calculated as the ratio of weight of the right ventricle to that of the left ventricle plus the interventricular septum as described in earlier studies [18].

### Isolation of mouse PASMCs

Mouse PASMCs were derived from pulmonary arterioles using an iron oxide magnetic separation method as previously described [19], [20]. Ten mice were used for each group to isolate PASMCs.

### HPASMC proliferation assay

The proliferation of HPASMCs was assessed by flow cytometry as previously described [15], [17]. Briefly, the cell pellet was re-suspended in 200 µL of PBS and then transferred to a cold vial of methanol (80 µL) and vortexed immediately. Cells were kept in methanol at 4°C overnight for fixing. The cells were resuspended in buffer containing 50 µL PBS, 3.3 µL RNAse (30 mg/mL) and the mixture was incubated at 4°C for 5 minutes. 450 µL of FACS buffer (PBS+2% fetal bovine serum) and 25 µL of propidium iodide (PI,1 mg/mL) were then added. The cells were analyzed using flow cytometry following 30 minute incubation at room temperature.

### Annexin V staining

An annexin V-FITC assay (BD Biosciences Pharmingen, San Jose, CA, USA) was performed as per the manufacturer instructions. The cells were incubated in the presence of annexin V-FITC and PI for 15 min at 37°C. The labeled cells were analyzed by flow cytometry as described in earlier studies [21].

### Wound healing assay

Wound healing assay and migration were performed as described in previous studies [15]. Briefly, an artificial wound was created using a 1 ml pipette tip on confluent HPASMCs monolayer. Flourescent images were taken at 0 and 24 hrs to visualize migrated cells and wound healing. The number of cells migrating into the wound area was counted and compared between EZH2 and vehicle transfected cells as described [22].

### EZH2 Transfection

PASMCs were transfected using the 4D-Nucleofector system according to the manufacturer's protocol from Lonza (Walkersville, MD). HPASMCs were cultured until they reached a confluency of 70% and then transfected with a total of 4 µg of plasmid DNA. Transfection efficiency was determined after 48 hours of transfection by both real-time PCR and western blot analysis for EZH2.

### SDS-PAGE and Western blotting

HPASMCs were harvested, washed twice with ice-cold PBS and lysed with ice-cold RIPA lysis buffer (PBS, 1% NP40, 0.5% sodium deoxycholate, 0.1% SDS, 1 mM PMSF, 10 mg/ml leupeptin, and 1 mM sodium orthovanadate). Cell lysates were obtained by centrifugation at 12,000 RPM for 15 min, and the supernatant protein concentrations were determined with a Bicinchronoic Acid Assay kit (Pierce, Rockford, IL). Protein lysates were prepared for SDS-PAGE by adding 1/4 volume of 4× SDS-PAGE sample buffer (100 mM Tris-Cl, pH 6.8, 200 mM DTT, 4% SDS, 0.2% bromophenol blue, 20% glycerol) and heating the mixture at 95°C for 5 min. Thirty micrograms of the protein were separated via SDS-PAGE and transferred onto a polyvinylidene difluoride membrane by electrophoresis (Immobilon P; Millipore, Bedford, MA). After blocking with TBS Tween buffer (10 Mm Tris-HCl, pH 8.0, 150 mM NaCl, 0.05% Tween-20) containing 5% nonfat dry milk for 1 h at room temperature, the membranes were incubated overnight at 4°C with blocking solution containing the indicated antibody (diluted 1∶1,000–1∶2,000). Membranes were washed and incubated with a suitable HRP-conjugated secondary antibody (Cell Signaling Technology, Danvers, MA). HRP activity was detected using an ECL kit according to the manufacturer's instructions (Pierce, Rockford, IL) [21], [23], [24].

### Quantitive RT-PCR

The relative expression levels of EZH2, Calponin, Cyclin D3 (CD3) and Cyclin-dependent kinase 2 (CDK2) were determined by real time PCR analysis. Total mRNA was isolated using the Trizol method and clean up was performed according to the manufacters protocol using RNA easy kit from Qiagen. Equal amounts of RNA were converted into cDNA using the iScript cDNA synthesis kit (Bio-rad, Hercules, CA). Real-time PCR downstream processing was performed on a Biorad cfx 96 real time PCR system using specific primers to EZH2, Calponin, CD3 and CDK2 as shown below. All primers were purchased from Integrated DNA Technologies. Inc (Coralville, IA) and reconstituted according to the manufacturer's instructions. The final concentrations of the primers used in the real time assays were 100 mM. Cq values were used to calculate the relative fold difference in mRNA levels compared with the normalized controls. All experiments were performed in triplicates. Primers used were: EZH2 5′- GCCAGACTGGGAAGAAATCTG-3′ (forward), 5′-TGTGCTGGAAAATCCAAGTCA-3′ (Reverse); Calponin 5′-GGCATCATTCTTTGCGAATTC-3′ (forward), TGGTGATGGCCTTGATGAAG (Reverse); **CDK2** - 5′- GCT TTC TGC CAT TCT CAT CG -3′(forward), 5′- GTC CCC AGA GTC CGA AAG AT -3′ (Reverse); **Cyclin D3** 5′- TGG ATG CTG GAG GTA TGT G -3′ (forward), 5′- CGT GGT CGG TGT AGA TGC -3′(Reverse); and GADPH-5′-CGGAGTCAACGGATTTGGTCGTAT-3′ (forward), 5′-AGCCTTCTCCATGGTGGTGAAGAC-3′ (Reverse).

### Chromatin Immunoprecipitation (ChIP) assay

The ChIP assay was performed on HPASMCs transfected with either EZH2 or vector control using the EpiTect ChIP one day kit (Qiagen, Valencia, CA) according to the manufacturer's instructions. Cells (10^7^) were fixed with formalin and sonicated after neutralizing with glycine. Immunoprecipitaion was performed using rabbit anti-tri-methyl histone (H3K27me3) antibody (Millipore, Billerica, MA) or with normal rabbit IgG. DNA in the eluted fractions were purified using spin columns provided in the kit and subjected to PCR using the following primers designed to amplify the promoter region of the calponin gene. (Calponin forward, GGGTGAGATCTGGCTGGGTA calponin reverse, GATCCTGGGTCTCACCCTCC). PCR using primers for myogenic differentiation 1 (MYOD1) promoter (Qiagen, Valencia, CA), a gene known to be regulated by histone methylation is used as a positive control.

### Statistical Analysis

All experiments were done in triplicates. A paired t-test was used to determine the statistical significance. A p-value of p<0.05 was accepted as statistically significant.

## Results

### Increased expression of EZH2 in PASMCs was associated with PAH progression in hypoxia-induced PAH mouse model

**Figure 1:**
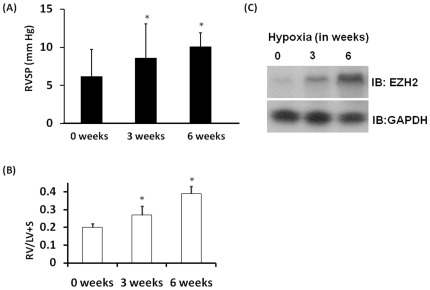
Increased expression of EZH2 in PASMCs was associated with PAH progression in hypoxia-induced PAH mouse model. Five age and sex-matched C57BL/6 mice were exposed to room air (21% FiO2) or to hypoxia (10% FiO2) at sea level. Averaged (A) RVSP and (B) RV/LV+S in mice at serial time points (0, 3 and 6 weeks) under hypoxic conditions of 10% oxygen (ten readings per mouse over 1 h, 20 mice per group at each time point). (C) EZH2 protein expression by western blot at serial time points under hypoxic conditions of 10% oxygen. All experiments were repeated at least 3 independent times ^*^ *P*<0.05 compared with 0 hrs.

An increased expression of EZH2 has been shown to correlate with proliferation of cancer cells, but its relevance in PAH is not known. To understand the relevance of EZH2 in PAH, mice were exposed to hypoxic conditions for 6 weeks to induce PAH. RVSP (mm Hg) and RV/LV+S were measured after 0, 3 and 6 weeks. Mice exposed to hypoxic conditions showed time dependent increase in RVSP and RV/LV+S indicating progression of PAH as shown in Figure 1A and 1B. At the same time points, PASMCs were isolated and proteins were probed for EZH2 as described in Materials and Methods. EZH2 levels were increased in hypoxia-induced PAH mice when compared to controls (Fig. 1C). These results suggest that increased expression of EZH2 in mouse PASMCs were associated with PAH progression in hypoxia-induced PAH mouse model. The increase in EZH2 levels along with the increase in PAH during hypoxia suggests an unidentified potential role for this protein in PAH.

### EZH2 expression is induced in proliferating HPASMCs

**Figure 2:**
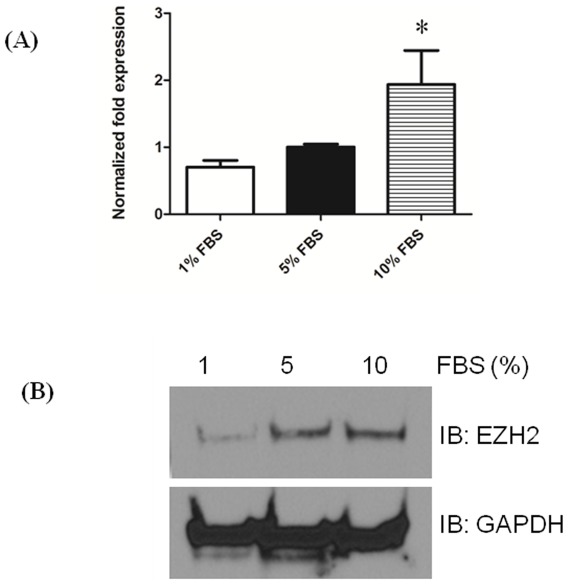
EZH2 expression induced in proliferating HPASMCs. HPASMCs were serum starved (1% FBS treated) or 10% serum treated for 24 h, and then (A) EZH2 expression was detected by qRT–PCR. (**B**) EZH2 protein expression was analyzed in serum-starved or 10% serum-treated HPASMCs by Western blotting. All experiments were repeated at least 3 independent times. ^*^ *P*<0.05 compared with 5% FBS.

To identify the potential role of EZH2 in PASMCs proliferation and PAH, we examined the expression of EZH2 in quiescent and proliferative HPASMCs by using quantitative real-time PCR and Western blot analysis. HPASMCs were treated with increasing concentrations of FBS (1%, 5% and 10%) to stimulate proliferating conditions and levels of EZH2 were analyzed by both real-time PCR and western blots. There was a 2 fold increase in EZH2 mRNA levels in proliferating HPASMCs in comparison to cells in normal growth media with 5%FBS, while the levels reduced in serum-starved PASMCs (Fig. 2A). A similar trend was also observed at the protein level illustrated in Figure 2B. This indicates that levels of EZH2 increases in response to proliferating conditions in HPASMCs.

### EZH2 overexpression induces proliferation of HPASMCs

**Figure 3:**
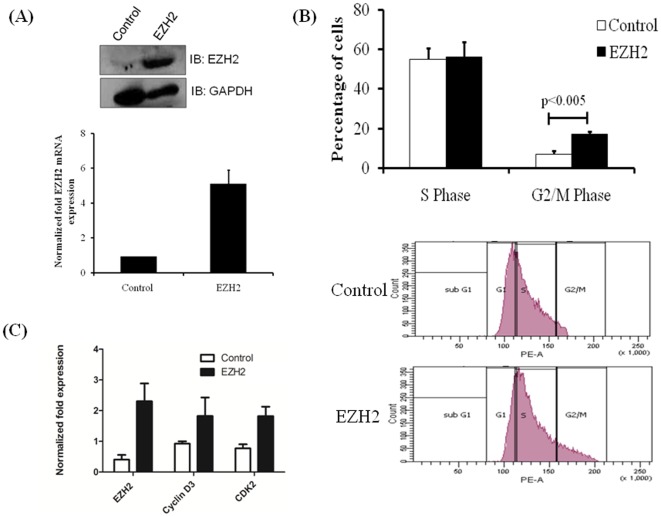
EZH2 overexpression induces proliferation of HPASMCs. HPASMCs were transfected with a plasmid expressing EZH2 or control plasmids using 4D-Nucleofector system. A) EZH2 protein expression was analyzed by western blot (upper panel) and mRNA expression was analyzed by real-time PCR (lower panel) to determine the transfection efficiency. B) The transfected cells were fixed and labeled with PI after 48 hrs and analyzed for cell cycle by flow cytometry. Percentage number of cells in S and G2/M phase of the cell cycle is plotted (upper panel) and representative histograms from the cell cycle analysis of EZH2 and control GFP plasmid transfected cells are shown (lower panel). C) Real-time PCR analysis of cell proliferation markers expressed as normalized fold expression is shown. All experiments were repeated at least 3 times and graphs shown are average of three independent experiments.

The observation that EZH2 content was high in SMC under proliferating conditions indicated that EZH2 could play a role in SMC proliferation, as is suggested in some cancerous cells. The role of EZH2 in HPASMCs proliferation was investigated by transfecting HPASMCs with plasmids expressing EZH2. Positive transfection was assessed by Western blotting and real-time PCR for EZH2 mRNA (Fig. 3A). The transfected cells were fixed and labeled with PI after 48 hrs and analyzed by flow cytometry to determine proliferation. The percentage of cells in the G2/M phase was significantly higher in EZH2-transfected cells in comparison to mock-transfected cells (Fig. 3B). There was no significant difference in the percentage of cells in the S-Phase of the cell cycle. As the percentage of cells in G2/M phase indicates proliferating cells, it can be inferred that the EZH2-transfected cells have a 1.5 fold higher rate of proliferation when compared with the control (mock-transfected) cells. Since there was an increase in number of dividing cells in EZH2 transfected HPASMCs, we further analyzed various proliferation markers by real-time PCR in EZH2 and mock transfected cells. There was an increase in CD3 and CDK-2 in EZH2 transfected cells compared to controls (Fig. 3C) indicating increasing progression of cell cycle and increased proliferation in EZH2 transfected HPASMCs. These findings support the hypothesis that EZH2 enhances HPASMCs proliferation.

### EZH2 overexpression induces migration of HPASMCs

**Figure 4:**
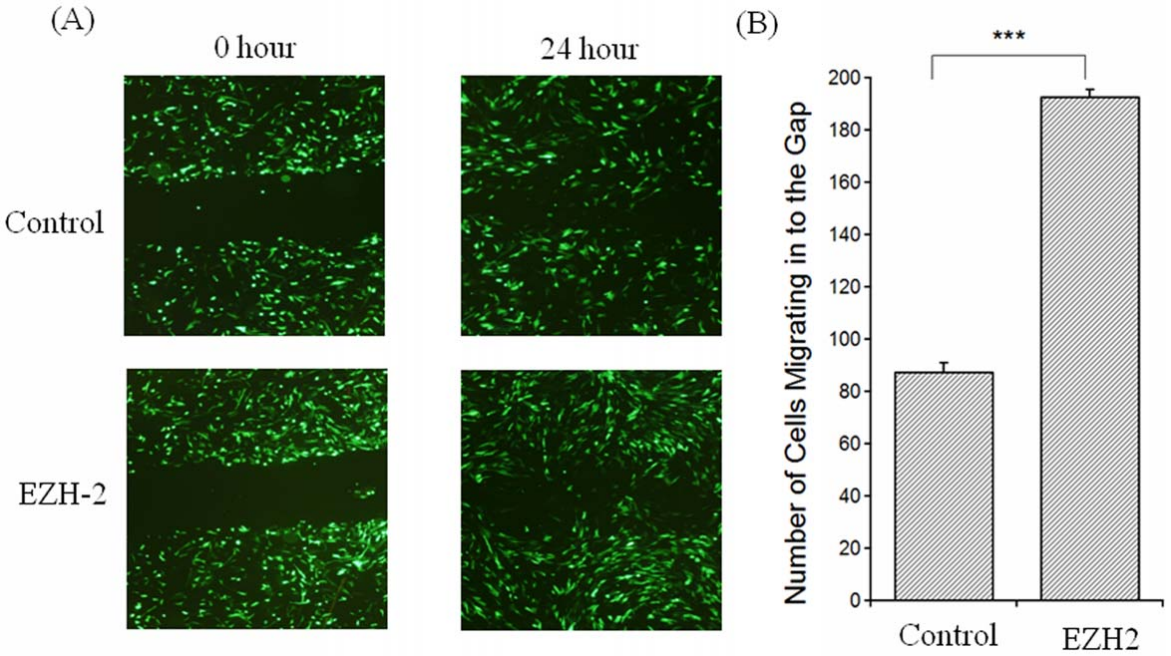
EZH2 overexpression markedly increases cell migration in HPASMCs as measured by wound healing assay. The HPASMCs were transfected with EZH2 and GFP plasmid or only with GFP plasmid (Control). An artificial wound was created using a 1 ml pipette on a confluent monolayer of cells. A) Images were taken at 0 and 24 hrs after wound. B) Migration was quantified visually as the number of cells migrating in to the gap and average of three experiments is plotted.

Vascular remodeling is a key feature of PAH and migration of proliferating SMCs is considered an important event of this remodeling process. The effect of EZH2 was assessed in migration of the proliferating HPASMCs by wound healing assays (Fig. 4A). There was a significant (p<0.001) increase in the wound healing in EZH2-transfected HPASMCs when compared to GFP-transfected controls (Fig. 4B) indicating that EZH2 not only enhances proliferation but also increases its migratory potential thus contributing to the pathogenesis of PAH.

### EZH2 overexpression reduces apoptosis in HPASMCs

**Figure 5:**
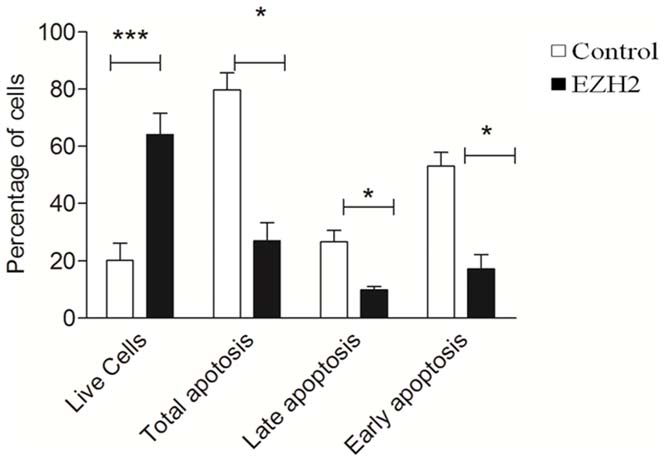
EZH2 overexpression reduces apoptosis in HPASMCs. HPASMCs cells were transfected with a plasmid expressing EZH2 or with GFP using 4D-Nucleofector system. Apoptosis was measured in transfected cells by using Annexin V staining. Propidium iodide (PI) incorporation was measured by flow cytometry to assess the mode of cell death. Relative fluorescence units represent the intensity of annexin V incorporation and PI incorporation was quantified. The noted experiments are representative of a minimum of four similar evaluations. ^*^ *P*<0.05 compared with control.

EZH2, apart from inducing cell proliferation, is also known to reduce apoptosis. Hence apoptosis was assessed by Annexin-V staining in HPASMCs transfected with EZH2 and in control (mock-transfected) HPASMCs. The apoptotic and necrotic cells could be differentiated by PI counter staining. EZH2 transfected cells have a significantly higher percentage (p<0.005) of live cells (64%), compared to the control group (23%) (Fig. 5), and transfected cells showed a 2.5 fold reduction in apoptotic cells when compared to mock-transfected cells (p<0.05). EZH2-transfected cells displayed a significant decrease in the percentage of cells in early and late apoptosis compared to control cells (p<0.05) (Fig. 5). These findings show that EZH2 overexpression leads to a significant decrease in apoptotic signaling in proliferating HPASMCs.

### EZH2 overexpression modulates the HPASMC phenotype

**Figure 6:**
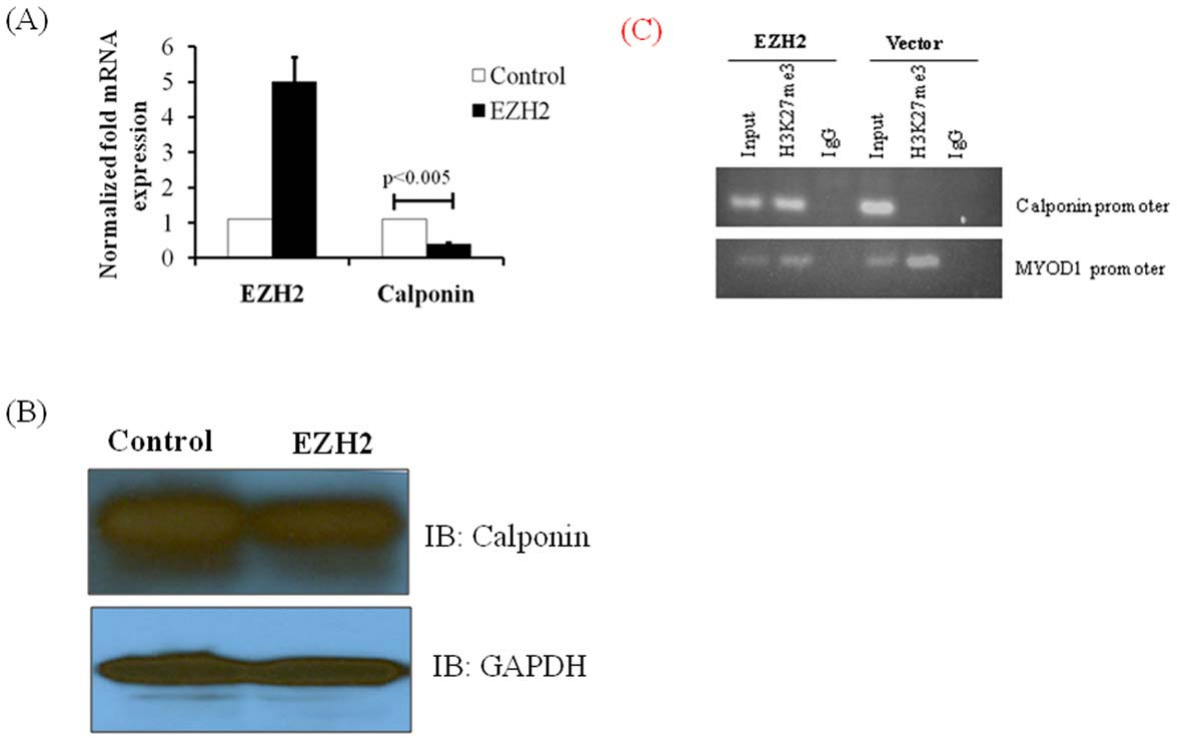
EZH2 overexpression modulates HPASMC phenotype. The HPASMCs were transfected with EZH2 or control plasmid and expression levels of calponin, a SMC phenotypic marker was assessed for A) mRNA by real-time PCR and B) protein levels by Western blot. All experiments were repeated at least 3 times and graph plotted is an average of three independent experiments. P<0.005 compared to controls. C) EZh2 increases binding to calponin-1 promoter in HPASMCs. DNA fragments interacting with H3K27me3 were pulled down by anti H3K27me3 or normal rabbit antibody. The presence of Calponin promoter DNA was checked by PCR using calponin promoter specific primers (upper panel). MYOD1 promoter segment binding to H3K27me3 was used as a positive control (lower panel). Calponin promoter region was amplified in EZH2 transfected cells and immunoprecipitated with anti-H3K27me3 but not in vector transfected cells (lanes 2 and 5). The immnoprecipitation with normal rabbit IgG did not yield any PCR amplification (lanes 3 and 6).

Dedifferentiated SMCs demonstrate accelerated proliferative phenotype that plays a critical role in PAH. Dedifferentiated SMCs show suppressed expression of calponin, a contractile phenotype marker. Calponin is a thin filament-associated protein that apparently functions as a negative regulatory element for SMC contraction. It may also have more broad cellular activities independent of contractility [25]. Calponin levels increase almost synchronously during the final stages of SMC differentiation [26]. Hence, during the late embryo stage and in adult tissues, calponin is used as a reliable marker for differentiated SMCs [25], [27].The established phenotypic and differentiation marker, calponin, was measured to determine whether EZH2 affects the SMC phenotype. There was a significant (p<0.005) suppression of calponin mRNA (Fig. 6A) and protein expression (Fig. 6B) in EZH2-transfected HPASMCs when compared to vehicle-transfected controls. These results indicate that EZH2 overexpression modulates HPASMCs to a more proliferative synthetic phenotype. The ChIP assays were performed in HPASMCs transfected with EZH2 or vehicle control to determine if EZH2 was directly suppressing calponin expression by histone trimethylation (H3K27me3). PCR amplification of the promoter region for calponin in ChIP assay with EZH2 transfected cells indicated that EZH2 was directly down regulating calponin expression by promoter compaction (Fig. 6C). These results indicate that EZH2 over expression modulates HPASMCs to a more proliferative synthetic phenotype.

## Discussion

PAH is a fatal disease caused by the obstruction of the small pulmonary arteries by vascular proliferation and remodeling. It is characterized by elevated pulmonary arterial pressures (PAP), and increased pulmonary vascular resistance leading to right-sided heart failure and death [1], [7]. Evidence reveals that epigenetic alterations may be involved in PAH such as changes in the phenotype or in gene expression states, including chromatin remodeling, DNA methylation and histone modifications [28]. The importance of EZH2 in the proliferation of HPASMCs and its implications in the development of PAH is demonstrated for the first time. EZH2 is a histone H3 lysine 27 methyltransferase of polycomb-repressive complex 2 [29]. EZH2 transcriptionally silences cohorts of developmental regulators in stem/progenitors and cancer cells [30].

PAH and cancer cells share similar proliferating phenotypes [31] [32] [33], and the overexpression of EZH2 is directly responsible for the de novo suppression of multiple genes in human cancer [14], [29], [30], [34] and is a known pathogenic factor in breast and prostate cancer [34]. An increased level of expression of EZH2 in hypoxia-induced PAH mice is consistent with this observation. Proliferating HPASMCs were also shown to have an increased expression of EZH2 compared to non-proliferating HPASMCs. Further studies on HPASMC-overexpressing EZH2 showed enhanced proliferation rates and confirmed the role of EZH2 in HPASMC proliferation. This observation is consistent with earlier observations in cancer cells suggesting a role for EZH2 in the control of proliferation in a variety of other normal, immortalized and transformed cell types [14], [30], [34], [35]. Vascular remodeling is a key feature of PAH and several studies reported that migration of proliferating vascular smooth muscles is an important event in the remodeling process [4], [6]. EZH2 knockdown suppress the invasion and migration of human epithelial ovarian cancer cells [15]. Consistent with this observation, the wound healing assays done in this study suggest that EZH2 enhances the migratory capabilities of proliferating HPASMCs thus playing a key role, not only in proliferation, but also in the migration of HPASMCs. Our results also indicate that EZH2-transfected HPASMCs are more resistant to apoptosis when compared to mock-transfected controls. Consistent with this observation is a report wherein EZH2 directly regulates the apoptotic process in cancer cells through epigenetically modulating pro-apoptotic Bim expression [36].

SMCs are not terminally differentiated and possess the ability to modulate their phenotype in response to external stimuli. SMCs convert from a sessile, “contractile” phenotype to a motile, proliferative “synthetic” phenotype during PAH [26], [37]. This change in phenotype is marked by a decrease in calponin, a SMC phenotype marker [25]. To check the effects of EZH2 transfection on the HPASMC phenotype, calponin levels were measured by both real-time PCR and Western blot analysis. There is a decrease in expression of calponin in EZH2 transfected cells indicating a shift in the SMC phenotype from a contractile to more proliferating one, which is a prominent feature of PAH. This phenotypic modulation of SMC is accompanied by accelerated migration and proliferation. However, at this point, we do not know how EZH2 contributes to the suppression of calponin expression. Over-expression of calponin in cultured SMCs suppresses their proliferation [38]. However, the definitive role of this molecule in SMCs proliferation is unclear. Therefore, it is important to understand whether EZH2 directly modulates calponin levels to drive SMCs to a more proliferative phenotype or whether its reduction of calponin is a result of de-differentiation of SMCs. ChIP assays were performed in HPASMCs over expressing EZH2 and for the first our results show that calponin is regulated by histone methylation by EZH2 in HPASMCs.

The fundamental mechanism of PAH is suggested to evolve from an imbalance between proliferation and anti-proliferation of PASMCs [2], [6]. However, the underlying causes or genetic factors responsible for this imbalance are not clear. This report indicates that EZH2 modulates proliferation, migration and apoptosis in HPASMCs and is increased in hypoxia-induced PAH. The molecular mechanisms of the modulation of these functions by EZH2 are not clear and a detailed study is warranted to further elucidate the importance of EZH2 in vascular smooth muscle cell function and in the development of PAH. This may help to develop new therapeutic targets to treat PAH.

In conclusion, EZH2 appears to be a key regulator of the PASMC function in the pulmonary vasculature. Elevated levels of EZH2 facilitate the proliferation and migration of PASMCs and enhance their resistance to apoptosis, leading to the thickening of the medial layer of pulmonary artery. These results suggest that EZH2 is a potent regulator of PASMC and a disturbance in its expression levels can lead to the development of PAH.
