# Correction: Enhancer of Zeste Homolog 2 Induces Pulmonary Artery Smooth Muscle Cell Proliferation

**DOI:** 10.1371/annotation/8580b50a-1556-4c86-aeb6-a88af839297a

**Published:** 2012-08-23

**Authors:** Salman A. Aljubran, Ruan Cox, Prasanna Tamarapu Parthasarathy, Gurukumar Kollongod Ramanathan, Venugopal Rajanbabu, Huynh Bao, Shyam S. Mohapatra, Richard Lockey, Narasaiah Kolliputi

The seventh author's name was spelled incorrectly. The correct name is: Shyam S. Mohapatra.

